# Cardiovascular Diseases of Developmental Origins: Preventive Aspects of Gut Microbiota-Targeted Therapy

**DOI:** 10.3390/nu13072290

**Published:** 2021-07-01

**Authors:** Chien-Ning Hsu, Chih-Yao Hou, Wei-Hsuan Hsu, You-Lin Tain

**Affiliations:** 1 Department of Pharmacy, Kaohsiung Chang Gung Memorial Hospital, Kaohsiung 833, Taiwan; cnhsu@cgmh.org.tw; 2 School of Pharmacy, Kaohsiung Medical University, Kaohsiung 807, Taiwan; 3 Department of Seafood Science, National Kaohsiung University of Science and Technology, Kaohsiung 811, Taiwan; chihyaohou@webmail.nkmu.edu.tw; 4 Department of Food Safety/Hygiene and Risk Management, College of Medicine, National Cheng Kung University, Tainan 701, Taiwan; whhsu@mail.ncku.edu.tw; 5 Department of Pediatrics, Kaohsiung Chang Gung Memorial Hospital and Chang Gung University College of Medicine, Kaohsiung 833, Taiwan; 6 Institute for Translational Research in Biomedicine, Kaohsiung Chang Gung Memorial Hospital and Chang Gung University College of Medicine, Kaohsiung 833, Taiwan

**Keywords:** aryl hydrocarbon receptor, cardiovascular disease, hypertension, gut microbiota, postbiotics, short chain fatty acid, prebiotics, probiotics, trimethylamine-N-oxide, developmental origins of health and disease (DOHaD)

## Abstract

Cardiovascular diseases (CVDs) can originate from early life. Accumulating evidence suggests that gut microbiota in early life is linked to CVDs in later life. Gut microbiota-targeted therapy has gained significant importance in recent decades for its health-promoting role in the prevention (rather than just treatment) of CVDs. Thus far, available gut microbiota-based treatment modalities used as reprogramming interventions include probiotics, prebiotics, and postbiotics. The purpose of this review is, first, to highlight current studies that link dysbiotic gut microbiota to the developmental origins of CVD. This is followed by a summary of the connections between the gut microbiota and CVD behind cardiovascular programming, such as short chain fatty acids (SCFAs) and their receptors, trimethylamine-N-oxide (TMAO), uremic toxins, and aryl hydrocarbon receptor (AhR), and the renin-angiotensin system (RAS). This review also presents an overview of how gut microbiota-targeted reprogramming interventions can prevent the developmental origins of CVD from animal studies. Overall, this review reveals that recent advances in gut microbiota-targeted therapy might provide the answers to reduce the global burden of CVDs. Still, additional studies will be needed to put research findings into practice.

## 1. Introduction

Cardiovascular diseases (CVDs), a cluster of disorders of the heart and blood vessels, are the leading cause of death worldwide [1]. Despite many advances in drug treatment, the prevalence of CVDs has continued to rise. Of note, most CVDs affect older adults. However, CVDs can originate from early life, from not only infancy and childhood, but tracing back to the prenatal stage as well (and gradually progressing across one’s life span) [2]. The cardiovascular system is one of the first body systems to appear within the fetus, which is vulnerable to the adverse intrauterine exposure to environmental insults [2]. The developmental origins of health and disease (DOHaD) theory posits that exposure to various insults during critical periods in fetal development leads to structural changes and functional adaption, resulting in increased risk of adult diseases, including CVDs [3]. Important support for the DOHaD concept came from epidemiological reports following birth cohorts in severe famines, which demonstrated that malnutrition during gestation induced a cluster of CVD risk factors, such as hypertension, dyslipidemia, obesity, kidney disease, type 2 diabetes, and cardiovascular morbidity in adult offspring [4,5]. Additionally, an observational study evaluating 2302 mother–child dyads reported that a better gestational CV health score in the mothers was associated with better CV health in the offspring from ages 10 to 14 years [6].

CVD risk can be shaped by a number of modifiable environmental risk factors often linked to diet. Diet influences the host’s health status by modulating the composition of the gut microbiota. Emerging evidence supports that gut microbiota may indirectly or directly influence cardiovascular risk [7,8,9]. Gut microbiota derived metabolites can act as a mediator of microbial influence through circulation on various target organs, including the cardiovascular systems [7,8]. Thus far, scientists have proposed several mechanisms by which dysbiotic gut microbiota contributes to CVD, such as alterations of microbiota-derived metabolite short-chain fatty acids (SCFA), increases of trimethylamine-N-oxide (TMAO), inhibition of nitric oxide (NO), and aberrant activation of the renin-angiotensin system (RAS) [7,8,9,10,11]. A meta-analysis study summarized 19 studies with 19,256 participants and found that individuals with high concentrations of TMAO and its precursors were associated with increased risks of major adverse cardiovascular events and all-cause mortality [12]. Maternal nutritional insults have been shown to alter gut microbiota balance, resulting in an increased risk of adult diseases [13]. However, relatively little is known about whether (and how) various early life insults could affect gut microbiota, resulting in CVDs in adult offspring.

Nevertheless, developmental programming, besides determining the risk for CVDs in adulthood, also offers an innovative approach to prevent CVDs by so-called reprogramming [14]. By switching therapy from adulthood to early life before disease occurs, we have the potential to postpone or reduce undesirable programming processes that would lead to CVDs. Over the year, the gut microbiota has gained more attention because, unlike non-modifiable CV risk factors, it can be modified through agents that modulate the intestinal bacterial flora, including prebiotics, probiotics, synbiotics, etc. [7,15,16,17]. Accordingly, one may assume that early gut microbiota-targeted therapy may serve as a reprogramming strategy to prevent the developmental origins of CVD.

The purpose of our scoping review is to provide insight on gut microbiota implicated in the developmental programming of CVD. Hence, we examine mechanisms linking gut microbiota to cardiovascular programming that, in light of the available evidence, can be considered therapeutic targets. In particular, we focus on addressing probiotics, prebiotics, and postbiotics as a reprogramming strategy for prevention of developmental programming of CVD.

We searched the PubMed/MEDLINE databases for studies published in English between January 1980 and May 2021, using the following search terms: “cardiovascular disease”, “developmental programming”, “DOHaD”, “reprogramming”, “gut microbiota”, “probiotics”, “prebiotics”, “synbiotics”, “postbiotics”, “mother”, “pregnancy”, “gestation”, “offspring”, “progeny”, “atherosclerosis”, “heart”, “vascular”, “endothelial dysfunction”, “stroke”, “thrombosis”, “aryl hydrocarbon receptor” and “hypertension”. Additional studies were then selected and assessed based on fitting references in eligible papers. We found that there are more than 3000 publications related to gut microbiota and CVD-related disorders. However, only approximately 10% belong to the research of DOHaD. Among them, hypertension accounts for nearly 80% of searchable publications.

## 2. Developmental Programming of CVD: Human Evidence

Epidemiological studies have consistently indicated that people exposed to famine during early life have disclosed strong relationships with CVD risk factors, such as obesity, dyslipidemia, hypertension, and type 2 diabetes [4,18,19]. Additionally, extensive evidence indicates that other early life adverse influences contribute to CVD in later life, including maternal illness, pregnancy complications, medication use in pregnancy, and in utero exposure to environmental pollutants [20,21,22]. In one example, the offspring of a diabetic pregnancy conveyed high risks for type 2 diabetes and obesity in adulthood—both risk factors are associated with CVD [23]. Prior research also suggested an association between medication use in pregnant women, such as glucocorticoid [24] and non-steroidal anti-inflammatory drugs [25], and adverse cardiovascular renal outcomes in their progeny. Several other risk factors in early life affecting cardiovascular outcomes in offspring have been reported, such as vitamin D deficiency [26], gestational hypertension [27], short-term breastfeeding [28], and excessively rapid weight gain postnatally [29]. Moreover, early life toxic environmental exposure, such as endocrine-disrupting chemicals, is linked to cardiometabolic traits in childhood [30]. The association between low birth weight (LBW), a clinical marker of inappropriate fetal growth, and later cardiovascular risk is another line of evidence for the developmental origins of CVD [31,32,33]. Previous studies on twins have reported that the lighter twins developed endothelial dysfunction and arterial narrowing and were more likely to die from ischemic heart disease [32,33].

However, epidemiological studies often do not provide information in which a direct cause–effect relationship can be established. Instead, the results from animal models can help one understand which developmental stage is critical for cardiovascular programming, to discover molecular mechanisms underlying the developmental origins of CVD, and to develop novel reprogramming strategies.

## 3. Implications of Gut Microbiota in the Developmental Origins of CVD 

### 3.1. Animal Models Related to Gut Microbiota

Animal models have provided compelling evidence that adverse early life conditions induce cardiovascular programming, coinciding with altered gut microbiota. Table 1 summarizes animal studies documenting the association between gut microbiota, early life insults, and subsequent CVDs in adult offspring [34,35,36,37,38,39,40,41,42,43,44,45,46,47,48,49,50,51]. CVDs often occur after a prolonged asymptomatic phase in childhood. For the sake of brevity, the present review is solely restricted to data obtained from adult offspring.

A variety of environmental insults in early life were reported to induce cardiovascular programming related to alterations of gut microbiota, including maternal high-fructose diet [34,35,36], maternal high-fructose diet plus 2,3,7,8-tetrachlorodibenzo-p-dioxin (TCDD) exposure [37], maternal chronic kidney disease [38], maternal minocycline administration [39], maternal asymmetric dimethylarginine (ADMA), trimethylamine N-oxide (TMAO) exposure [40], maternal hypertension [41,42], maternal high-fat and/or post-weaning high-fat diet [43,44,45,46,47], maternal N^G^-nitro-L-arginine-methyl ester (L-NAME) administration plus post-weaning high-fat diet [48], maternal Western-style diet [49], maternal dyslipidemia [50], and prenatal androgen exposure [51].

The most common cardiovascular outcome being studied in altered gut microbiota-related animal models is hypertension [34,35,36,37,38,39,40,41,42,45,46,47,48,50,51], followed by endothelial dysfunction [44] and ventricular hypertrophy [51]. Abnormalities in gut microbiota were also related CV risks, such as obesity [43,44,49,51], insulin resistance [43,45], increased lipid profile [45,50], and nonalcoholic fatty liver disease [44,48]. Abnormalities in gut microbial richness and diversity have been linked to a higher risk of developing other CVDs, such as coronary artery disease, cardiomyopathy, and heart failure [52]. However, little information exists regarding the role of gut microbiota in the developmental origins of those CVDs.

As Table 1 illustrates, the rat is the most common species used. The possible reason might be that rat models provide a low-cost option with a short life cycle that is easy to handle [53,54]. Although other species, such as rabbits, sheep, and pigs, are used in DOHaD research [53,54], limited information is provided on using large animals to study gut microbiota and the developmental origins of CVD.

In adulthood, every one month of the rat is equivalent to three human years [55]. Table 1 lists the timing of cardiovascular outcomes determined from 12 weeks to 4 months of age in rats, which corresponds to humans from childhood to early adulthood. Accordingly, knowledge gaps about long-term adverse effects of early life insults on CVD and gut microbiota in older adult offspring remain large.

### 3.2. Potential Mechanisms Underlying the Developmental Origins of CVD

**Figure 1:**
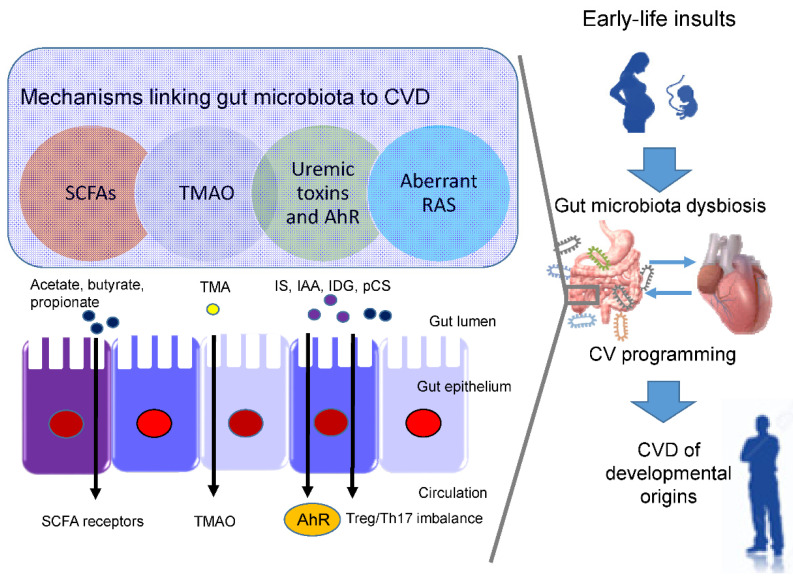
Overview of the common mechanisms linking gut microbiota dysbiosis to cardiovascular programming. CVD—cardiovascular disease. SCFA—short chain fatty acid. TMAO—trimethylamine N-oxide. AhR—aryl hydrocarbon receptor. RAS—renin-angiotensin system. Treg—T regulatory cells. TH17—T helper 17 cells. IS—indoxyl sulfate. IAA—indoleacetic acid. IDG—3-indoxyl β-d-glucuronide. pCS-p—Cresyl sulfate. TMA—trimethylamine.

In view of the fact that a variety of insults during fetal development generates similar cardiovascular outcomes (e.g., hypertension) in adulthood, this raises the possibility that a common pathway is involved in the pathogenesis of the developmental origins of CVD. Presently, several mechanisms have been linked to the developmental origins of CVD, such as oxidative stress, nitric oxide (NO) deficiency, dysregulated nutrient-sensing signals, gut microbiome dysbiosis, epigenetic regulation, sex differences, etc. [2,14,20,21,22,54]. Some of them are interrelated to gut microbiota dysbiosis. Recent evidence has been accumulated by deciphering the role of gut microbiota in the developmental origins of CVD, including alterations of SCFAs and their receptors, increases of TMAO, uremic toxins, and aryl hydrocarbon receptor (AhR), and aberrant renin-angiotensin system (RAS) [10,11] (Figure 1). Each of them will be discussed in turn.

### 3.3. SCFAs and Their Receptors

Short chain fatty acids (SCFAs) are formed during bacterial fermentation of carbohydrates in the gut. SCFAs have one to six carbon atoms (C1-C6), mainly consisting of acetate (C2), propionate (C3), and butyrate (C4) [56]. SCFAs are generally known to induce vasodilatation, in favor of antihypertension [56].

In spontaneously hypertensive rats (SHRs), hypertension is related to decreased abundance of acetate- and butyrate-producing bacteria [57]. SCFAs exert their regulation on BP mainly by activating their SCFA receptors, including G protein-coupled receptor 41 (GPR41), 43 (GPR43), 109A (GRP109A), and olfactory receptor 78 (Olfr78) [56]. The Olfr78 is expressed in the renal juxtaglomerular apparatus, where it mediates renin secretion in response to SCFAs to elevate BP [58]. In addition to hypertension, the metabolism of SCFAs is also a factor contributing to obesity [59].

In a maternal minocycline exposure model, minocycline-induced hypertension is associated with decreased plasma levels of acetate and butyrate [39]. Another report demonstrated that maternal garlic oil therapy protects against increases in blood pressure induced by high-fat diets in adult male rat offspring, which coincided with increases of acetate, butyrate, and propionate levels, as well as their producing microorganisms [60]. Additionally, SCFA supplementation in pregnancy and lactation have shown positive benefits on hypertension of developmental origins [35,61]. These findings support the notion that SCFAs and their receptors might be a key mechanism underlying developmental programming of hypertension.

### 3.4. TMAO

Emerging evidence indicates that a high TMAO level correlates with CVD mortality [62]. Besides CVD, TMAO also contributes to other cardiovascular risks, such as chronic kidney disease (CKD), type II diabetes, insulin resistance, and NAFAD [63]. TMAO can activate p38 mitogen-activated protein kinase (MAPK) signaling, nuclear factor-κB (NF-κB) signaling, inflammatory gene expression, and leukocyte-endothelial cell adhesion in atherosclerosis development [64]. TMAO is a small colorless amine oxide produced by gut microbiota metabolism. Its production is a two-step process. The first step involves gut microbial trimethylamine (TMA) formation from dietary precursors (e.g., choline and carnitine); in the second step, TMA is oxidized to TMAO by flavin-containing monooxygenases (FMOs) in the liver [65]. TMAO is then either excreted by the kidney or transported to the tissues.

Maternal TMAO administration can induce hypertension in adult male offspring [40]. Conversely, 3,3-dimethyl-1-butanol (DMB, a TMA inhibitor) treatment during pregnancy and lactation protected adult offspring against hypertension programmed by a maternal high-fructose diet, which was associated with the reduction of TMA and TMAO levels [35]. Moreover, perinatal resveratrol therapy prevented maternal CKD-induced hypertension in adult male rat offspring and was associated with a decreased TMAO-to-TMA ratio [38]. These observations suggest a pathogenic association between the TMA-TMAO pathway and the developmental origins of CVD.

### 3.5. Uremic Toxins and Aryl Hydrocarbon Receptor

Uremic toxins play an important role in cardiovascular morbidity and mortality in patients with CKD [66]. One example is TMAO, a cardiovascular risk factor and uremic toxins. Additionally, several tryptophan metabolites are known as gut microbiota-derived uremic toxins, such as indoxyl sulfate (IS), indoleacetic acid (IAA), and indoxyl--D glucuronide (IDG) [67]. These tryptophan-derived uremic toxins have proinflammatory, procoagulant, prooxidant, and pro-apoptotic effects, all of which are implicated in the pathogenesis of CVD [68]. Among them, IS and IAA were related to the risk for cardiovascular morbidity and mortality in uremic patients [69]. In CKD, IS can activate monocytes, intensify inflammatory process, augment oxidative stress and impair hemostatic system, all of which are major contributors to the development of CVD [70,71].

Moreover, IAA and several microbial tryptophan catabolites are ligands for aryl AhR [72]. AhR signaling modulates pro-inflammatory T helper 17 (TH17) axis and triggers inflammation, by which activation of AhR by its ligands is closely associated with the development of CVD [73,74]. An imbalance of T regulatory cells (Treg) and TH17 cells is involved in the development of hypertension [75]. In patients with CKD, Treg/Th17 imbalance has been associated with the pathogenesis of cardiovascular complications [76].

In a maternal CKD-induced hypertension model, maternal tryptophan therapy preventing offspring hypertension was associated with mediation of the AhR signaling pathway [77]. On the other hand, antagonizing AhR signaling by resveratrol has been reported to protect adult offspring against hypertension in several hypertension models of developmental origins [36,78,79]. Prior research demonstrated that IS and AhR both can promote thrombosis [80,81]. As thrombus formation, secondary to atherosclerotic plaque disruption, plays a major role in the development of several CVDs [82], attention will need to be paid to better understand the interplay between AhR and uremic toxins underlying developmental programming of thrombotic-related CVDs.

Another uremic toxin is p-Cresyl sulfate (pCS), derived from aromatic amino acids metabolized by gut bacteria [83]. Increased levels of pCS have been related to worsening CV outcomes in patients with CKD [83]. Previous work demonstrated that p-Cresyl sulfate could increase expression of proinflammatory cytokines and adhesion molecules, therefore mechanistically promoting atherogenesis [84].

### 3.6. RAS

The RAS is a complex network that is implicated in CVD [85]. Within the RAS, regulation is achieved through a cascade of proteases that generate several bioactive peptides [86]. The classical RAS is composed of angiotensin-converting enzyme (ACE), angiotensin (ANG) II, and angiotensin II type 1 receptor (AT1R). Activation of the classical RAS triggers vasoconstriction and inflammation under pathophysiological conditions, thus promoting hypertension and cardiovascular damage [85]. Emerging evidence suggests that aberrant RAS plays a key role in cardiovascular programming and RAS-based interventions can be used as a reprogramming strategy to prevent DOHaD-related disorders [86]. Treating young offspring with renin inhibitor aliskiren [87], ACE inhibitor captopril [88], or angiotensin receptor blocker (ARB) losartan [89] between 2 and 4 weeks of age was shown to protect against hypertension programmed by various maternal insults in adulthood.

Angiotensin-converting enzyme 2 (ACE2), a homologue of ACE, converts ANG II to ANG-(1–7) that negatively regulates the RAS [90]. Via regulation of intestinal amino acid transport, previous research showed that ACE2 plays a crucial non-catalytic role in gut biology and modulation of gut microbiota [91]. One previous study reported that administration with the ACE2 activator or with ANG-(1–7) during pregnancy could alleviate cardiovascular dysfunction in adult SHR offspring [92]. Another report showed that the antihypertensive effect of probiotics might be due to their ability to produce ACE inhibitory peptides [93].

Since gut microbiome dysbiosis has been connected to CVD by modulating the gut RAS [94], these findings support the notion that the interplay between gut microbiota and the RAS implicates the pathogenesis of cardiovascular programming, although this remains speculative.

## 4. Preventing the Developmental Origins of CVD by Gut Microbiota-Targeted Therapy

### 4.1. Gut Microbiota-Targeted Therapy

As mentioned, gut microbiota is closely related to the developmental origins of CVD. Researchers have gradually turned their attention to focus on gut microflora and related metabolites as a potential target for therapeutics [95,96]. Probiotics are live microorganisms, which, when administered in adequate amounts, confer a health benefit to the host [97]. Prebiotics can promote the growth of probiotics and inhibit the growth of pathogen [97]. Synbiotics refer to dietary supplements, combining probiotics and prebiotics. A substantial body of research supports that prebiotics and/or probiotics supplementation have possible benefits for the prevention and management of CVD [8,15,95,96].

Additionally, fecal microbial transplantation (FMT) is being extensively studied in microbiome-associated pathologies, such as CVD [98]. However, its potential application in CVD remains limited, mainly as it brings both beneficial and harmful bacteria to the patients, which may lead to adverse complications.

Another gut microbiota-targeted modality is the use of microbial derived metabolites, namely postbiotics. Postbiotics include any substance produced or leased through the metabolism of the gut microbes, which have a positive effect on the host [99]. As postbiotics do not contain live microorganisms, they appear to lack serious side effects while maintaining similar effectiveness to probiotics [99].

**Figure 2:**
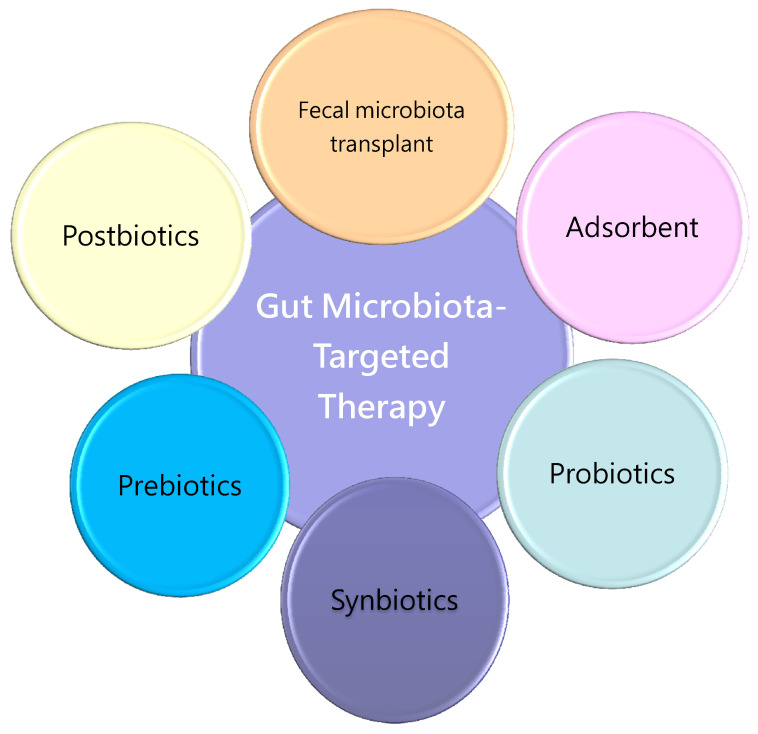
Schema outlining the potential gut microbiota-targeted therapies as a reprogramming strategy to prevent cardiovascular disease of developmental origins.

Moreover, microbiota-derived uremic toxins can be reduced by AST-120, an oral charcoal adsorbent [100]. In patients with CKD, AST-120 treatment has shown cardiovascular benefits [101,102]. However, the influence of adsorbents on gut microbiota compositions, and their related metabolites in other CVD populations instead of CKD, remains largely unknown. We propose the notion of the schema summary to address the potential gut microbiota-targeted therapies as a reprogramming strategy involved in the the developmental origins of CVD, which is illustrated in Figure 2.

### 4.2. Uses of Probiotics and Prebiotics in Pregnant Women

In clinical practice, the most commonly used gut microbiota-targeted modalities are probiotics and prebiotics. When discussing the therapeutic benefits of probiotics and prebiotics in clinical utility, particular attention should be paid to their safety. Human studies regarding probiotic supplementation during pregnancy are limited [103]. Probiotic supplementation for pregnant women is generally safe and may have a protective role in preeclampsia [104], gestational diabetes [105], vaginal infections [106], and spontaneous preterm delivery [107]. Likewise, little is known about the use of prebiotics in pregnant women. One report demonstrated that supplementation with indigestible oligosaccharide prebiotics increased the number of maternal fecal *Bifidobacterium* spp., while this bifidogenic effect may not be transferred to the neonatal gut [108]. Although results from human studies on probiotics supplementation in the treatment of maternal conditions during pregnancy are beneficial, essentially no information exists regarding the effectiveness in protecting offspring against adult diseases.

### 4.3. Uses of Probiotics and Prebiotics in Newborn and Infants

In humans, microbiota colonization evolves continuously after birth, and by the age of three, the microbiota resembles adult-like composition [109]. Accordingly, manipulation of infant gut microbiome, through prebiotic and probiotic supplementation, may provide opportunities to promote health in later life [110]. Breast milk is the primary source of oral feeding. With prebiotic and probiotic properties, breast milk is crucial for the establishment of health-promoting microorganisms and plays specific effects on infantile immune function [111].

Despite the use of probiotics being considered safe in healthy infants and children, the effect of probiotics on the developing immune system and safety in neonates is largely unknown. Likewise, adverse effects of prebiotics added to infant formula are rarely documented. However, uncertainties remain about the doses and species necessary to confer beneficial effects, duration of supplementation, and the long-term effects of prebiotic and probiotic supplementation in neonates and infants.

### 4.4. Reprogramming Strategy for the Developmental Origins of CVD

Due to ethical considerations in humans, experiments to develop reprogramming interventions, targeting gut microbiota to prevent the developmental programming of CVD, have provided compelling evidence in animal models.

As mentioned in Table 1, various early life insults can alter the offspring’s gut microbial composition, leading to adverse CV outcomes. Conversely, early intervention targeting on gut microbiota has shown benefits against cardiovascular programming. Although novel gut microbiota-targeted modalities are tested in established CVD, few of them have been examined in early life for the developmental origins of CVD.

Table 2 summarizes studies that document microbiota-based reprogramming interventions in animal models, of cardiovascular programming, restricting those applying to critical periods during early development [34,35,37,39,47,112,113]. Moreover, we merely considered studies reporting CV outcomes with long enough follow-up of the offspring.

As shown in Table 2, gut microbiota-based interventions have been dominated by experiments using rats. Available gut microbiota-targeted treatment modalities used as reprogramming interventions include probiotics, prebiotics, and postbiotics.

Although several probiotic microorganisms are shown to benefit cardiovascular health [15], there is still limited information regarding their role on the developmental origins of CVD. Supplementation with *Lactobacillus casei* throughout gestation and lactation periods protected adult male rat offspring against hypertension induced by the maternal high-fructose diet [34] or high-fat diet [47]. Another study demonstrated that maternal *Lactiplantibacillus plantarum WJL* treatment prevented cardiovascular dysfunction programmed by the maternal high-fat and high-cholesterol diet [45].

So far, only oligofructose and inulin have been investigated in regards to their reprogramming effects in the developmental origins of CVD [34,47,112], despite several types of prebiotics providing various health benefits [114]. Maternal oligofructose therapy attenuated hepatic steatosis and insulin resistance induced in adult offspring born to dams received high-fat/-sucrose diets [112]. Using a high-fat model [47], our prior research reported that inulin treatment protected against hypertension in adult rat offspring, coinciding with alterations of gut microbiota, and particularly increased the proportion of *Lactobacillus*, a well-known probiotic strain. Similarly, inulin supplementation during gestation and lactation protected adult offspring against hypertension programmed by the maternal high-fructose diet, which was related to an increased plasma propionate level [35].

Since SCFAs are products of fermentation of polysaccharides by gut microbiota, they have been used as postbiotics for CVD [115]. Although acetate, butyrate, and propionate have been shown to reduce BP in animal models of hypertension [116,117], only maternal acetate supplementation was reported to protect offspring hypertension programmed by a high-fructose diet [35]. Another example of postbiotics use in the developmental origins of CVD is conjugated linoleic acid [113]. In humans, conjugated linoleic acid production is performed by gut microbiota, mainly by the Bifidobacterium species [118].

In addition to metabolites, postbiotics include many different constituents, such as microbial cell fractions, extracellular polysaccharides, extracellular vesicles, functional proteins, cell lysates, cell wall-derived muropeptides, etc. [115]. However, essentially no information exists regarding the use of other types of postbiotics in the developmental origins of CVD.

There are other gut microbiota-related interventions applied to preventing the developmental origins of CVD. DMB, a structural analogue of choline, is able to inhibit microbe-dependent TMA and host TMAO formation. At least two studies have reported that maternal DMB therapy protected hypertension in adult offspring born to mothers exposed to high-fructose diets [35] or high-fructose diets plus TCDD exposure [37].

Of note, there are prebiotic-like components commonly found in consumable functional foods, such as flavonoids, polyphenols, and vitamins. Take resveratrol, a polyphenolic compound found in grapes and wine, as an example. A growing body of studies firmly supports that the beneficial effects of resveratrol on CVD are related to its prebiotic effect on gut microbiota [119,120]. As we reviewed elsewhere, early resveratrol therapy could be a reprogramming agent to protect against the developmental origins of CVD [121]. One study demonstrated that maternal resveratrol therapy protected against maternal CKD-induced hypertension in adult progeny, which was associated with restoration of microbial richness and diversity, and increased abundance of *Lactobacillus* and *Bifidobacterium* [38]. In another study, using a maternal L-NAME plus high-fat diet model, maternal resveratrol therapy protected against hypertension of developmental origins, coinciding with reduction of the *Firmicutes* to *Bacteroidetes* ratio, a microbial marker for hypertension [10,11]. Likewise, prebiotic-rich food, such as garlic, was used as used as a strategy to reprogram and prevent high-fat diet-induced hypertension in adult progeny [60]. Therefore, there is an urgent need to gain a greater understanding of other prebiotic-like components implicated on the developmental origins of CVD.

## 5. Conclusions and Perspectives

Previous studies have indicated that early gut microbiome and microbial metabolites might influence cardiovascular programming and exert adverse cardiovascular effects in later life. This review sought to highlight the value of gut microbiota-targeted interventions if applied early, to treat (and help prevent) CVD.

At face value, it would be logical to consider early life probiotics or prebiotics supplementation in potential reprogramming interventions in regards to the developmental origins of CVD. However, there are many aspects still unsolved. At a deeper level, little reliable information currently exists regarding the reprogramming effects of gut microbiota-targeted interventions in human trials. Future work in large prospective trials is needed to better identify (and appreciate) probiotic species and improve formulation of prebiotics for the developmental origins of CVD.

Animal studies suggest certain prebiotics may contribute to the prevention of the developmental origins of CVD, although the exact mechanisms have not been fully elucidated. What is missing from the literature is a greater understanding of whether the uses of numbers of prebiotic-like components or prebiotic-rich food in pregnancy and lactation can also alter gut microbiota and derived metabolites to prevent offspring against the developmental origins of CVD.

Due to the complex nature of postbiotics, the long-term effects of early life postbiotics interventions in various models of cardiovascular programming, either individually or in combination, are incomplete, or are awaiting further clarification. In exception for definitions provided by the International Scientific Association for Probiotics and Prebiotics (ISAPP), and the Food and Agriculture Organization of the United Nations–World Health Organization (FAO–WHO) for prebiotics and probiotics [122,123], so far there is a lack of a clear definition for postbiotics. A clear definition is important from a regulatory perspective, as postbiotics can be made with various probiotic species via a wide range of inactivation methods.

In conclusion, gut microbiota is a meaningfully pathogenetic link in the developmental origins of CVD. After all of this remarkable growth in gut microbiota-based interventions and greater understanding of cardiovascular programming, we expect that translating this emerging body of evidence into clinical practice is a future strategy that could reduce the global burden of CVD.

## Figures and Tables

**Table 1:** Eligible animal models reporting cardiovascular outcomes related to alterations of gut microbiota.

Animal Models	Cardiovascular Outcomes	Programming Mechanisms Related Gut Microbiota	Species/ Gender	Age at Measure	Ref.
Maternal high-fructose diet	Hypertension	Decreased SCFA receptor GPR41 and GPR43 expression	SD rat/M	12 weeks	[34]
Maternal high-fructose diet	Hypertension	Decreased plasma TMA level; reduced phylum *Verrucomicrobia* and genus *Akkermansia* abundance	SD rat/M	12 weeks	[35]
Maternal plus post-weaning high-fructose diet	Hypertension	Decreased abundance of genera *Bacteroides, Dysgonomonas*, and *Turicibacter*	SD rat/M	12 weeks	[36]
Maternal high-fructose diet and TCDD exposure	Hypertension	Increased abundance of genus *Gordonibacter*	SD rat/M	12 weeks	[37]
Maternal adenine-induced chronic kidney disease	Hypertension	A decreased α-diversity and an increased F/B ratio; A decreased abundance of the genus *Bifidobacterium*	SD rat/M	12 weeks	[38]
Maternal minocycline administration	Hypertension	An increase F/B ratio, and decreased genera *Lactobacillus*, *Ruminococcus*, and *Odoribacter* abundance	SD rat/M	12 weeks	[39]
Maternal ADMA and TMAO exposure	Hypertension	Decreased abundance of family *Erysipelotrichaceae*			[40]
Maternal hypertension	Hypertension	An increased abundance of the genera *Bifidobacterium, Lactobacillus, Turicibacter*, and *Akkermansia*	SHR/M	12 weeks	[41]
Maternal hypertension	Hypertension	An increased F/B ratio	SHR/M	12 weeks	[42]
Maternal high-fat diet	Obesity and insulin resistance	Decreased gut microbiota richness	C57BL/6J mouse/M and F	12 weeks	[43]
Maternal high-fat diet	Obesity and nonalcoholic fatty liver disease	Decreased α-diversity	C57BL/6J mouse/M and F	17 weeks	[44]
Maternal high-fat and high-cholesterol diet	Hypertension, endothelial dysfunction, increased lipid profile and insulin resistance	Decreased α-diversity	Wistar rat/M	90 days	[45]
Maternal plus post-weaning high-fat diet	Hypertension	An increased F/B ratio; a reduction of genera *Lactobacillus* and *Akkermansia*	SD rat/M	16 weeks	[46,47]
Maternal L-NAME administration plus post-weaning high-fat diet	Hypertension	An increased F/B ratio	SD rat/M	16 weeks	[48]
Maternal Western-style diet	Obesity and nonalcoholic fatty liver disease	An increase in abundance of genus *Ruminococcus*	C57BL/6J mouse/M	20 weeks	[49]
Maternal dyslipidemia	Hypertension and increased lipid profile	A decrease of genera *Lactobacillus*	Wistar rat/M and F	24 weeks	[50]
Prenatal androgen exposure	Hypertension, decreased heart rate, obesity, and increased thickness of left ventricle	An increased abundance of bacteria associated with production of SCFAs.	Wistar rat/F	4 months	[51]

Studies tabulated according to animal models, and age at measure; SD rats—Sprague-Dawley rats; SHR—spontaneously hypertensive rat; M—male; F—female; TCDD—2,3,7,8-tetrachlorodibenzo-p-dioxin; ADMA—asymmetric dimethylarginine; TMAO—trimethylamine N-oxide; L-NAME—N^G^-nitro-L-arginine-methyl ester; F/B ratio—*Firmicutes* to *Bacteroidetes* (F/B) ratio.

**Table 2:** Summary of gut microbiota-targeted modalities used as reprogramming interventions for cardiovascular disease of developmental origins.

Gut Microbiota-Targeted Intervention	Animal Models	Species/Gender	Age at Evaluation	Reprogramming Effects	Ref.
Probiotics					
*Lactobacillus casei* 2 × 10⁸ CFU/day via oral gavage during pregnancy and lactation	Maternal high-fructose diet	SD rat/M	12 weeks	Prevented hypertension	[34]
*Lactobacillus casei* 2 × 10⁸ CFU/day via oral gavage during pregnancy and lactation	Perinatal high-fat diet	SD rat/M	16 weeks	Prevented hypertension	[47]
*Lactiplantibacillus plantarum WJL* 1 × 10⁸ CFU/day via oral gavage during pregnancy and lactation	Maternal high-fat and high-cholesterol diet	Wistar rat/M	90 days	Prevented cardiovascular dysfunction	[45]
Prebiotics					
5% *w*/*w* long chain inulin during pregnancy and lactation	Maternal high-fructose diet	SD rat/M	12 weeks	Prevented hypertension	[34]
5% *w*/*w* long chain inulin during pregnancy and lactation	Perinatal high-fat diet	SD rat/M	16 weeks	Prevented hypertension	[47]
10% *w*/*w* oligofructose during pregnancy and lactation	Maternal high-fat/-sucrose diet	SD rat/M	21 weeks	Attenuated hepatic steatosis and insulin resistance	[112]
Postbiotics					
Magnesium acetate 200 mmol/L in drinking water during pregnancy and lactation	Maternal high-fructose diet	SD rat/M	12 weeks	Prevented hypertension	[35]
1% conjugated linoleic acid during pregnancy and lactation	Maternal high-fat diet	SD rat/M	18 weeks	Prevented hypertension and endothelial dysfunction	[113]
Others					
1% DMB in drinking water during pregnancy and lactation	Maternal high-fructose diet	SD rat/M	12 weeks	Prevented hypertension	[35]
1% DMB in drinking water during pregnancy and lactation	Maternal high-fructose diet and TCDD exposure	SD rat/M	12 weeks	Prevented hypertension	[37]

Studies tabulated according to types of intervention, animal models, and age at evaluation. DMB-3,3—maternal dimethyl-1-butanol. TCDD—2,3,7,8-tetrachlorodibenzo-p-dioxin. SD—Sprague–Dawley rat.

## Data Availability

Data will be available upon request.
